# The Role of Flotillins in Regulating Aβ Production, Investigated Using *Flotillin* 1-/-, *Flotillin* 2-/- Double Knockout Mice

**DOI:** 10.1371/journal.pone.0085217

**Published:** 2014-01-21

**Authors:** Vassilis Bitsikas, Kirsi Riento, Jonathan D. Howe, Nicholas P. Barry, Benjamin J. Nichols

**Affiliations:** Laboratory of Molecular Biology, Medical Research Council, Cambridge, Cambridgeshire, United Kingdom; Cambridge University, United Kingdom

## Abstract

Flotillin 1 and flotillin 2 associate in the plasma membrane to form microdomains that have roles in cell signaling, regulation of cell-cell contacts, membrane-cytoskeletal interactions, and endocytosis. They are thought to be involved in the trafficking and hence processing of the Amyloid Precursor Protein, APP. In this study we set out to obtain *in vivo* confirmation of a link between flotillins and cleavage of APP to release amyloidogenic Aβ peptide, and to generate tools that would allow us to ask whether flotillins are functionally redundant. We used a mouse model for Aβ-dependent cerebral amyloidosis, APPPS1 mice, combined with deletion of either *flotillin 1* singly, or *flotillin 1* and *flotillin 2* together. There was a small but significant reduction in Aβ levels, and the abundance of congo-red stained plaques, in brains of 12 week old mice lacking *flotillin 1*. A similar reduction in Aβ levels was observed in the *flotillin 1-/-*, *flotillin 2-/-* double knockouts. We did not observe large effects on the clustering or endocytosis of APP in *flotillin 1-/-* mouse embryonic fibroblasts. We conclude that flotillins are likely to play some role in APP trafficking or processing, but the relevant cellular mechanisms require more investigation. The availability of *flotillin 1-/-*, *flotillin 2-/-* mice, which have no overt phenotypes, will facilitate research into flotillin function *in vivo*.

## Introduction

Proteolysis of the single pass trans-membrane protein APP (Amyloid Precursor Protein) leads to the generation of Aβ (beta amyloid), a 40 or 42 amino acid peptide that is the main constituent of the amyloid plaques found in the brains of people with Alzheimer's disease [1]. Sequential cleavage of APP by beta and gamma secretases generates the Aβ peptide, while the cleavage product of alpha and gamma secretases is not amyloidogenic. How the activities of the alpha, beta and gamma secretases are regulated spatially and temporally, and hence the amount of Aβ produced, are not completely understood (this large literature is reviewed in [1]–[3]). It is likely, however, that endocytosis and sub-cellular trafficking of APP contributes to its differential processing [4].

Flotillins are palmitoylated and myristoylated proteins that associate with the inner leaflet of the plasma membrane [5]–[7]. There are 2 flotillin paralogues in metazoans, flotillin 1 and 2. Association and oligomerisation of flotillin 1 and 2 leads to the formation of plasma membrane puncta, or microdomains, with a defined size [8], [9]. Both flotillins are required for formation of these structures [9], [10]. The function of flotillin microdomains is not fully understood, but they are likely to be important for linking the plasma membrane and the cytoskeleton [10]–[12], for signaling events [13], [14], for regulating cell-cell adhesion [15], and for membrane traffic during endocytosis [5].

Several observations link flotillins to APP trafficking. Flotillins accumulate in the endosomal system of neurons from a transgenic mouse model of amyloid plaque formation, and in the brains of humans with Alzheimer's disease [16], [17]. Increased Aβ production, moreover, causes intracellular accumulation of Aβ in flotillin-positive endosomes [18]. The intracellular domain of APP has been reported to bind to flotillins, and flotillins and APP may both bind to a common partner, LGI3 (leucine rich glioma inactivated 3) [19], [20]. Importantly, siRNA mediated knockdown of flotillin 2 impairs the endocytosis of APP, alters the nanometer-scale clustering of APP in the plasma membrane, and reduces Aβ production in tissue culture cell models [21]. One outstanding question relates to whether there is redundancy between the flotillins in terms of a functional relationship with APP, as different studies report a role for flotillin 1, and others flotillin 2 [19], [21].

In this study we have investigated the role of flotillin 1 in APP trafficking and processing. We have used cells from *flotillin 1* knockout mice to determine whether endocytosis or plasma membrane clustering of APP is affected by the complete absence of the flotillin 1 protein. We have assayed the *in vivo* role of flotillins in Aβ production, comparing both the amount of Aβ and the abundance of plaques in brains of a transgenic mouse model for Aβ plaque formation (APPPS1 mice, which overexpress human APPswe and L166P mutant presenilin 1 [22]), in the presence and absence of the *flotillin 1* gene. We find that *in vivo* there is a small but reproducible reduction in Aβ accumulation and hence plaque formation in the absence of *flotillin 1*. We tested whether there is functional redundancy between flotillin 1 and flotillin 2 *in vivo* by generating *flotillin 1-/-*, *flotillin 2-/-* double knockout mice. The amount of Aβ in *flotillin 1-/-* single knockouts and *flotillin 1-/-*, *flotillin 2-/-* double knockouts expressing the APPPS1 transgenes is the same. Our data confirm that flotillins are likely to have some link to the trafficking or processing of APP, and that the absence of one flotillin is unlikely to be compensated by the presence of the other flotillin protein, suggesting that flotillins probably form a complex to exert their function. However, the precise mechanism by which flotillins affect APP processing is still unclear.

## Results

**Figure 1:**
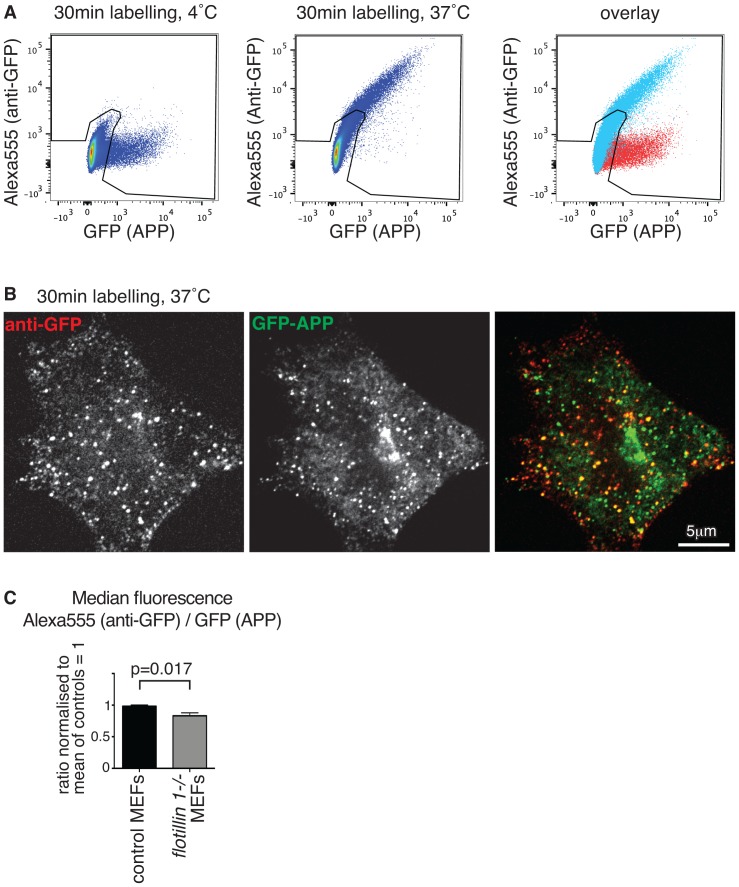
Deletion of *flotillin 1* causes a small reduction in APP endocytosis in mouse embryonic fibroblasts. All cells are primary MEFs **A**. Cells expressing GFP-APPswe were labelled on ice, or at 37°C with Alexa555 conjugated anti-GFP antibodies and subsequently analysed by flow cytometry. The right hand panel overlays signals from 4°C and 37°C. The difference between the signals is due to endocytosis of the antibody at 37°C. **B**. Colocalisation of GFP-APPswe with internalised anti-GFP(Alexa555) antibodies following 30 minute incubation at 37°C **C**. Primary MEFs from *flotillin 1-/-* mice or congenic controls, expressing GFP-APPswe, were labelled for 30 min at 37°C with Alexa555 anti-GFP antibodies as in A. In order account for variable expression levels, APP uptake was calculated as the ratio of median Alexa555 fluorescence (anti-GFP) over median GFP fluorescence, after gating for GFP-positive cells as shown by the black line overlaid in A. This ratio was normalised so that the mean of the control values equalled 1, to allow comparison of different experiments. Data from 5 separate flow cytometry experiments are shown. Bars are SEM.

We carried out experiments to determine whether flotillin 1 has a role in endocytosis of APP. MEFs (mouse embryonic fibroblasts) were transfected with a plasmid expressing GFP-APPswe (In all experiments we used a GFP-tagged version of the Swedish mutation of APP [23]). GFP is present on the extracellular domain of the protein. Endocytosis assays were based on flow cytometry analysis, thereby allowing measurement in high numbers of cells with a wide range of expression levels. When transfected cells were incubated with fluorescent anti-GFP antibodies on ice, only a small amount of antibody bound to the cells, consistent with low levels of full length APPswe at the plasma membrane, as APPswe is rapidly cleaved by secretases after exocytosis [4], [24]. Upon incubation at 37°C, however, the amount of antibody present in the cells progressively increased in transfected cells, but did not change in cells not expressing APPswe (Figure 1A). This specific and temperature dependent increase in signal is likely to reflect internalisation of antibody bound to GFP-APPswe. Consistent with this, confocal microscopy revealed that after incubation with anti-GFP antibodies at 37°C the antibody co-localised with GFP-APPswe in apparently intracellular, endosomal compartments of transfected cells (Figure 1B), but was not present in un-transfected cells. A clear positive correlation between the amount of overexpressed APP and the amount of antibody accumulating in the cells was evident in the flow cytometry data (Figure 1A). When the amount of antibody accumulation in control and *flotillin 1-/-* cells was quantified and compared, using FACS as in Figure 1A, there was a slight but significant reduction in the *flotillin 1-/-* cells (Figure 1C). The small magnitude of this effect could reflect an indirect role for flotillin 1 in APP endocytosis or intracellular trafficking.

**Figure 2:**
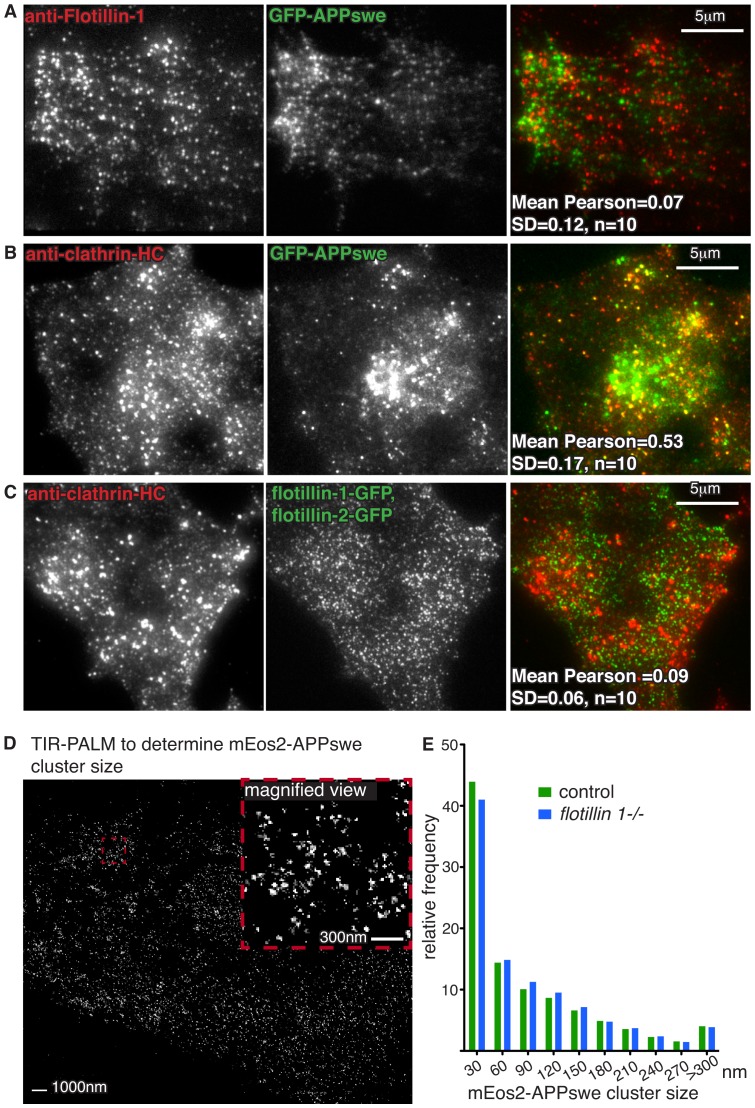
Deletion of *flotillin 1* does not alter clustering of APP in mouse embryonic fibroblasts. **A.** Immunofluorescence staining for endogenous flotillin-1, which resides in flotillin microdomains at the plasma membrane, does not overlap with GFP-APPswe in images acquired with TIR illumination. Pearson's correlation coefficient calculated from 10 similar images is shown. **B.** GFP-APPswe colocalised extensively with antibody against clathrin heavy chain, detected by indirect immunofluorescence and TIR illumination. Pearson's correlation coefficient calculated from 10 similar images is shown. **C.** Consistent with previous reports, no significant overlap was observed between flotillins and clathrin, detected as in B. Pearson's correlation coefficient calculated from 10 similar images is shown. **D.** PALM during TIR illumination was used to determine the size of mEos2-APPswe clusters at the plasma membrane of MEFs. A representative image after particle detection and reconstruction with QuickPALM is shown. Fitted centroids from the PALM analysis are represented as a single pixel. The intensity of that pixel is proportional to the accuracy of the fitted centroid, and the intensities of centroids fitted to the same pixel are summed. E. Frequency distribution of cluster size for mEos2-APPswe at the plasma membrane, comparing control and *flotillin 1-/-* MEFs. At least 10 images were analysed for each genotype.

In order to further investigate the relationship between flotillin 1 and APP endocytosis and trafficking, we carried out co-localisation experiments. Flotillin 1 did not co-localise to any significant extent with GFP-APPswe at the plasma membrane, when imaged using TIR (total internal reflection) illumination (Figure 2A). This, coupled with the small size of the effect of flotillin 1 deletion on APP uptake, argues against direct internalisation of APP in flotillin microdomains. Indeed, APP has previously been shown to be internalised via clathrin-coated pits [4], [24], and extensive co-localisation between GFP-APPswe and clathrin was observed (Figure 2B). It is possible that flotillins could act as some kind of specialized adaptor for specific recruitment of some cargoes to coated pits [5]. However, co-localisation between flotillins and clathrin was much less extensive than between GFP-APPswe and clathrin (Figure 2C), so it is unlikely that flotillins recruit APP to coated pits.

siRNA-mediated knockdown of flotillin 2 has been reported to perturb the clustering of APP in the plasma membrane [21]. We used PALM (photo-activation localisation microscopy [25]) during TIR illumination to determine the distribution of APP at the plasma membrane in *flotillin 1-/-* MEFs, and MEFs from congenic control mice. For visualisation of APP clusters we tagged the extracellular part of APPswe with monomeric EOS2 fluorescent protein [26]. As predicted by previous studies [24], mEOS2-APPswe was found in clusters at the plasma membrane, and we were able to resolve clusters as small as 30 nm in diameter (Figure 2D). There was, however, no difference detected in the size of these clusters between *flotillin 1-/-* and control cells (Figure 2E). Therefore neither co-localisation, nor super-resolution imaging data suggest a specific mechanism by which flotillin 1 could be involved in APP traffic.

**Figure 3:**
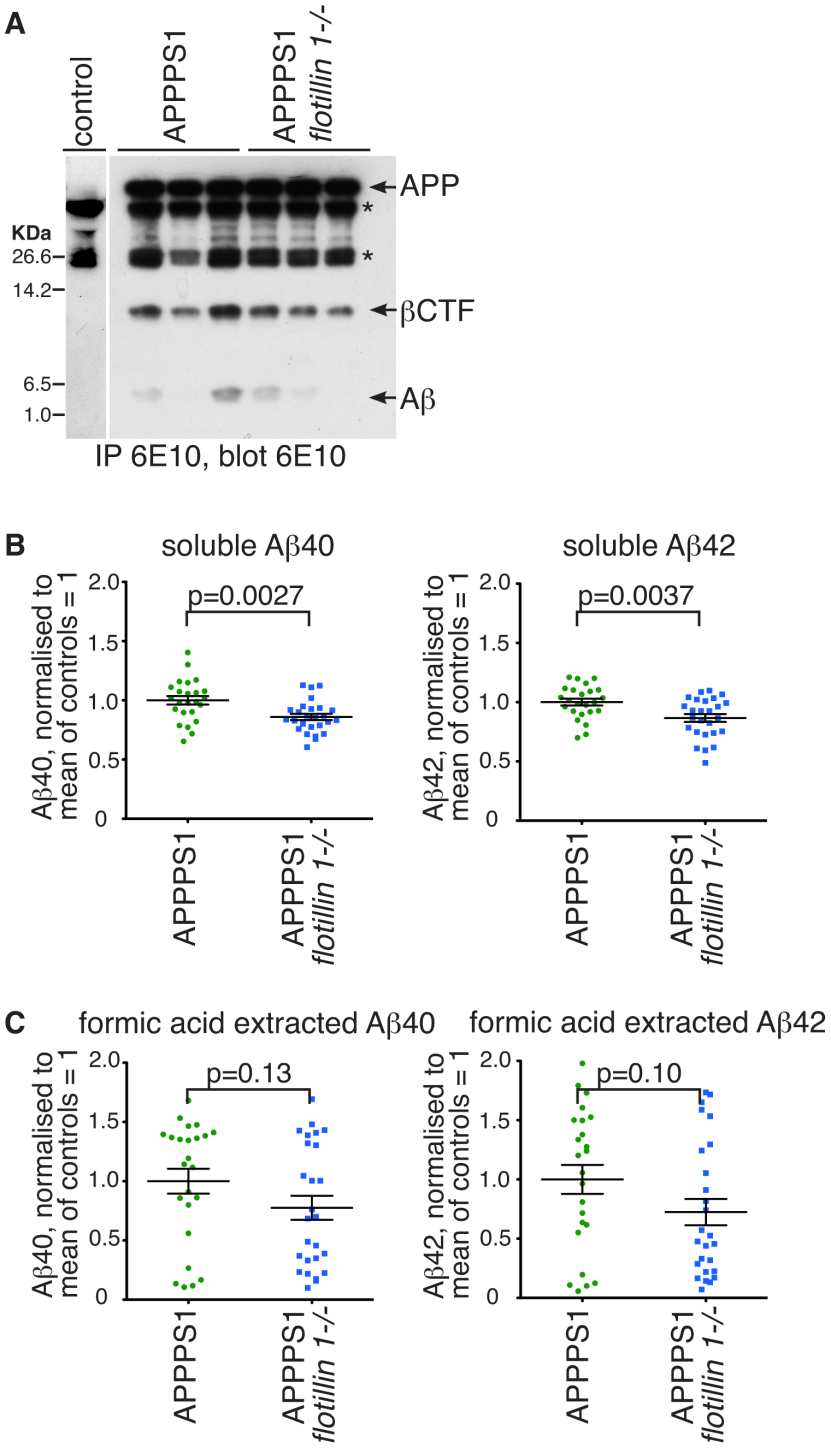
Deletion of *flotillin 1* reduces the accumulation of both soluble Aβ, and Aβ in formic-acid extractable plaques, in brains of APPPS1 mice. **A.** APP from RIPA buffer solubilised lysates of mouse brain was immunoprecipitated with the monoclonal antibody 6E10, and the precipitates analysed by Western blotting with the same antibody. The bands corresponding to full length APP (APP), β C-terminal fragment of APP (βCTF), and Aβ are indicated. Bands with an * are present in mice not expressing human APP, and are most likely antibody heavy and light chains. 3 mice of each genotype were analysed. Approximate positions of protein molecular weight markers are indicated. **B.** Brain tissues from 12 week old APPPS1 or APPPS1, *flotillin 1-/-* mice were harvested and Aβ levels were measured quantitatively using ELISA. Soluble Aβ40 and Aβ42 were present in the supernatant after tissue homogenisation and centrifugation at 20,000 rcf. Each data point represents assay from the brain of one mouse. Bars are SEM. **C.** Brain tissues were harvested as in B above, but Aβ40 and Aβ42 were extracted with 70% formic acid from the pellet, after tissue homogenisation and centrifugation, and the levels assayed using ELISA. Each data point represents assay from the brain of one mouse. P values were calculated using Student's t-test. Bars are SEM.

Aiming to explore the potential relationship between flotillin 1 and APP transport and processing in a more physiologically relevant system, we turned to *in vivo* experiments. APPPS1 mice, which coexpress the Swedish mutation of APP and L166P mutated presenilin 1, provide a tractable model for APP-dependent amyloidogenesis, developing Aβ plaques from 6–8 weeks after birth [22]. We crossed APPPS1 mice with *flotillin 1-/-* mice. Western blots of brain extracts using the 6E10 monoclonal against APP confirmed that full length human APP is expressed in the resultant mouse strains in equal amouts (Figure 3A). However, in these blots, the abundance of APP cleavage products varied from individual to individual (Figure 3A), and no clear difference between control and *flotillin 1-/-* mice was observed. As Western blotting did not yield clear-cut results, we elected to assay Aβ levels in control and *flotillin 1* knockout mice quantitatively using ELISA (enzyme linked immuno-absorbent assay), using commercial reagents for detection of the two different forms of Aβ of 40 and 42 amino acids [27]. Brain hemisphere extracts from 12 week old mice were prepared by homogenization and a 20,000 g spin (the supernatant from which was used to assay soluble Aβ), followed by extraction of Aβ from the pellet with 70% formic acid (formic acid solubilizes aggregated Aβ present mostly in amyloid plaques [28]). The amount of soluble 40 and 42 amino acid Aβ measured in brains from *flotillin 1-/-* mice was consistently less than that found in congenic controls (Figure 3B). The same effect was observed when formic acid extracted Aβ was assayed, although in this case the data were more variable (Figure 3C). These data demonstrate that flotillin 1 is likely to have an effect on Aβ levels, and hence potentially APP processing, *in vivo*.

**Figure 4:**
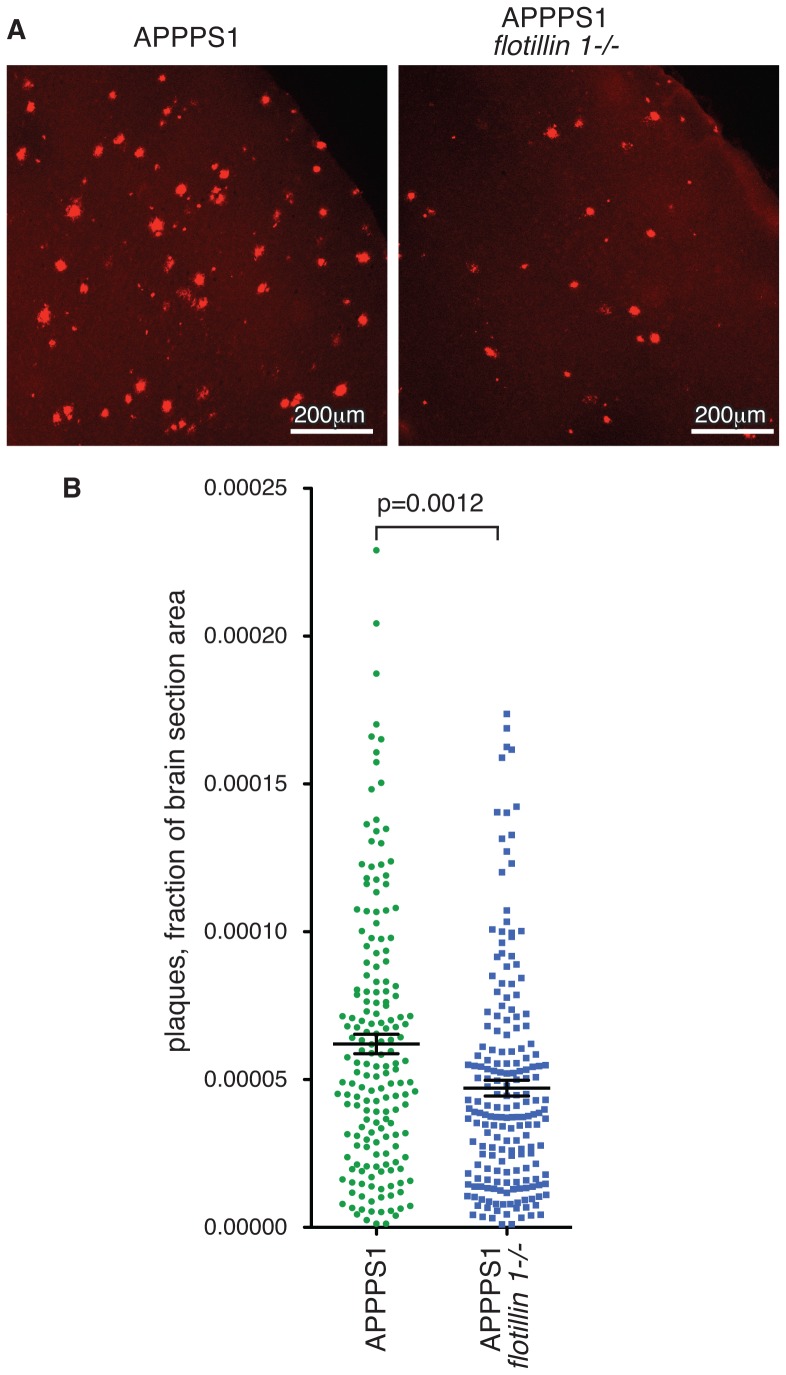
Deletion of *flotillin 1* reduces the accumulation of Congo Red stained plaques in brains of APPPS1 mice. **A.** Brain hemispheres from *flotillin 1-/-* and congenic control mice were sectioned on a cryotome, stained with Congo Red, imaged on a stereo microscope and subsequently analysed with ImageJ. **B.** Quantification of the proportion of the area of each brain slice stained with Congo Red. Each data point represents one slice, data are pooled from 10 *flotillin 1-/-* and 10 control mice. Bars are SEM.

In order to provide confirmation that flotillin 1 is involved in formation of Aβ plaques, we used Congo Red staining of 25 µm coronal sections of frozen brain hemispheres from *flotillin 1-/-* mice and congenic controls (Figure 4A). Congo Red is a metachromatic anionic dye that stains insoluble protein deposits, like amyloid plaques [29]. Quantification of the area of each section covered by plaques, in around 25 sections from each of 10 control and 10 knockout mice, revealed a significant decrease in plaque formation in the *flotillin 1* knockouts to 76% of that measured in the controls (Figure 4B). Therefore both use of Congo Red to stain plaques and ELISA assays for Aβ accumulation imply that flotillin 1 has a role in regulating cleavage of APP and hence amyloid plaque formation *in vivo*.

**Figure 5:**
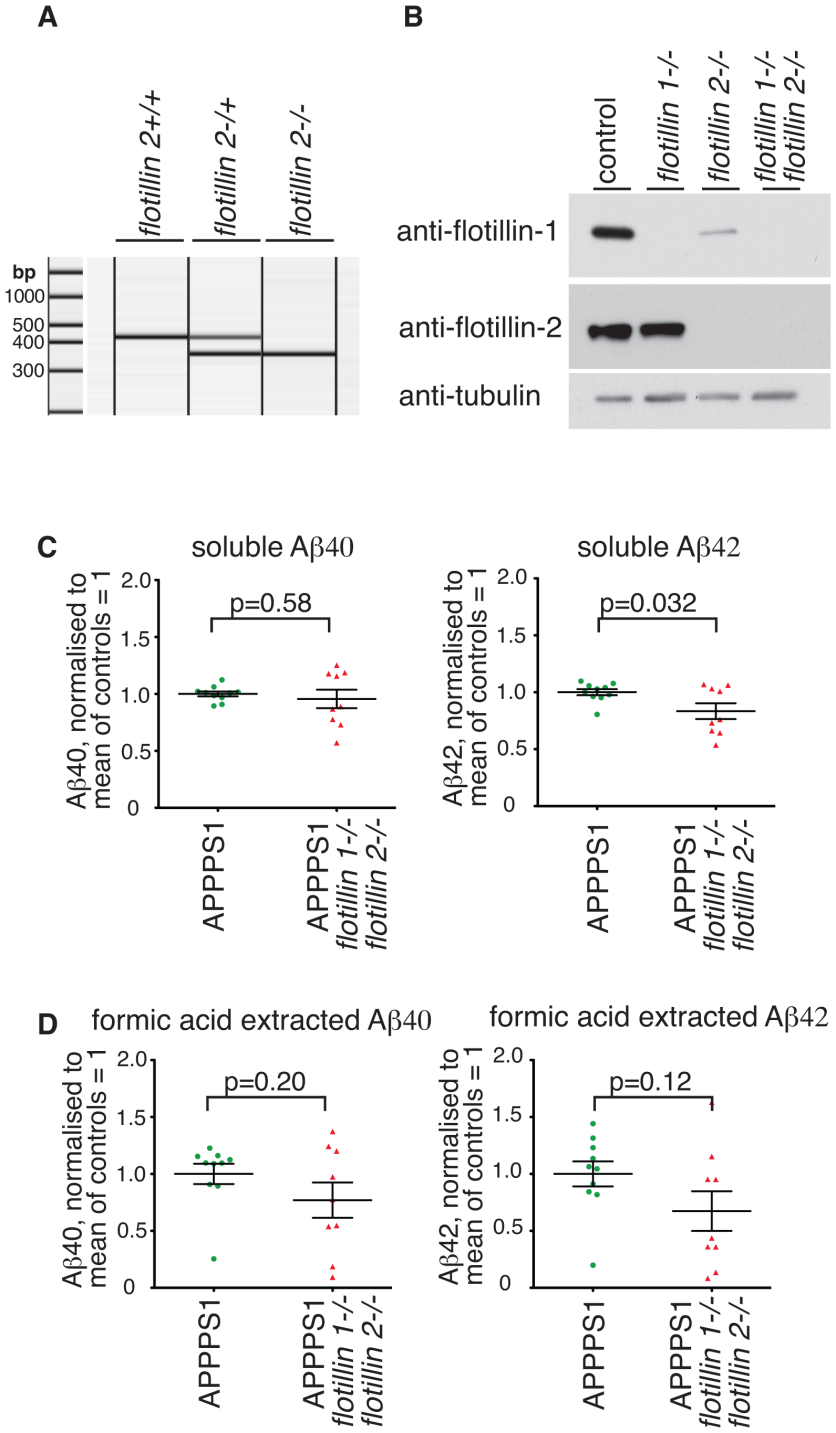
Deletion of *flotillin 1* and *flotillin 2* causes the same reduction in Aβ levels as is observed when flotillin 1 alone is deleted. **A.** The insertion of a gene trap in the *flotillin 2* locus causes a band shift from 500 bp to 350 bp in PCR-based genotyping. The left hand panel indicates position of DNA size markers. **B.** In *flotillin1 -/-* MEFs the amount of flotillin 2 is slightly reduced, while in *flotillin 2-/-* MEFs there is a dramatic reduction in the amount of flotillin 1 present. As expected, no flotillin proteins can be detected in double knockout cells. **C.** Brain tissues were harvested and Aβ levels were measured quantitatively using ELISA. Soluble Aβ40 and Aβ42 were present in the supernatant after tissue homogenisation and centrifugation at 20,000 rcf. Each data point represents assay from the brain of one mouse with the genotype shown. Bars are SEM. **D.** Brain tissues were harvested as in C above, but Aβ40 and Aβ42 were extracted with 70% formic acid from the pellet, after tissue homogenisation and centrifugation, and the levels assayed using ELISA. Each data point represents assay from the brain of one mouse. P values were calculated using Student's t-test. Bars are SEM.

One limitation of the experiments described thus far in this study is that flotillin 2 could compensate for the absence of flotillin 1. It has been reported that flotillin 2 has a more significant effect on APP endocytosis than flotillin 1 [21]. These experiments used siRNA depletion and it may be hard to compare the efficiency of functional depletion for different targets. Aiming to resolve these issues, and to ask whether there is redundancy between flotillin 1 and flotillin 2, we produced flotillin 2 knockout mice, using appropriately targeted embryonic stem cells from the Knockout Mouse Project (www.komp.org). Absence of flotillin 2 expression in these mice was confirmed by PCR (Figure 5A), and by Western blotting (Figure 5B). After back-crossing the *flotillin 2-/-* mice with C57Bl/6J mice for 4 generations, they were crossed with *flotillin 1-/-* mice to produce homozygous double knockouts, *flotillin 1-/-*, *flotillin 2-/-*. The double knockouts showed no gross or obvious abnormalities compared with congenic controls or either single knockout. In *flotillin 1* knockouts flotillin 2 expression was reduced, and residual flotillin 2 was no longer concentrated in membrane microdomains [10]. In *flotillin 2* knockouts flotillin 1 expression was less than 15% of control levels (Figure 5B), and in the double knockouts neither protein could be detected, as one would expect (Figure 5B).

After crossing the *flotillin 1-/-*, *flotillin 2-/-* mice with APPPS1, soluble and formic-acid extractable Aβ levels in the brain of 12 week old animals were assayed by ELISA exactly as previously. Again, there was a reduction in both pools of Aβ (Figure 5C, Figure 5D). In the case of formic acid extracted Aβ, variability in the data was too high to support the conclusion that this reduction was statistically significant, but the trend of reduced Aβ was apparent in all cases. Importantly, there was no significant difference between Aβ levels in the *flotillin 1-/-* mice and the double knockouts (compare Figure 3 and Figure 5: when Aβ levels in *flotillin 1-/-*, *flotillin 2-/-* double knockout and *flotillin 1-/-* mice were compared with each other, using Students T test, no significant difference was detected). Therefore there is no functional redundancy between flotillin 1 and flotillin 2, at least in terms of Aβ production.

## Discussion

Crossing of APPPS1 mice with *flotillin 1-/-* and *flotillin 1-/-*, *flotillin 2-/-* knockout mice has allowed us to provide evidence that flotillins are likely to be involved in some way in the regulation of APP processing and hence Aβ production. In brain from *flotillin 1-/-* and *flotillin 1-/-*, *flotillin 2-/-* mice expressing the APPPS1 transgenes we observed less Aβ and less amyloid plaques, as compared with controls. The magnitude of the reduction was, however, relatively small. We must, therefore, consider the possibility that flotillins are not directly involved in APP trafficking *per se*, but rather have some homeostatic role in a separate cellular process that impacts indirectly on Aβ production. The fact that our experiments on APP traffic and clustering in *flotillin 1* knockout MEFs did not reveal any strong phenotype is consistent with this idea.

Further experiments that address the molecular basis for flotillin function in different cellular processes are clearly needed. It is also possible that in the APPPS1 mouse, where Aβ production is being driven at high rates by ectopic expression of human APP and PS1 [22], the effects of deletion of flotillin genes are not the same as they would be in more physiological contexts. Therefore our results, although suggestive, should not be interpreted as completely ruling out an important or direct role for flotillins in APP traffic.

The issue of whether flotillins 1 and 2 are co-dependent in their cellular functions, or whether they can also function individually, has been unclear. There are several reports in the literature of siRNA experiments where knockdown of one flotillin, but not the other, has an effect [7], [13], [21], [30], [31]. On the other hand, both flotillins are required for formation of characteristic punctate membrane microdomains, and the large majority of both proteins is present in the protein complexes which define these microdomains [5], [8], [9], [11], [12]. Both flotillins have been found to be expressed at constant levels across 16 different human tissues, suggesting that the composition of the complexes should be uniform in all tissues [32]{Eisenberg, 2013 #453}{Eisenberg, 2013 #453}{Eisenberg, 2013 #453}. Our quantitative data on Aβ production allows us to state that, at least in this case, the phenotype of *flotillin 1-/-*, *flotillin 2-/-* double knockouts is no more severe than observed in *flotillin 1-/-* mice, so it is unlikely that the flotillin 2 protein present in the *flotillin 1-/-* mice is functioning in processes relevant to Aβ production.

The establishment of *flotillin 1-/-*, *flotillin 2-/-* double knockout mice will provide a powerful model system for investigating flotillin function without the complicating factor of potential functional redundancy between flotillins.

## Materials and Methods

### Transgenic mice

APPPS1 transgenic mice that co-express the Swedish mutation of APP and L166P mutated presenilin [22] were kindly provided by Michel Goedert and Isabel Lavenir (MRC-LMB). Flotillin 2 knockout mice were generated from Flot2 Gt(258D8)Cmhd embryonic stem cells, produced by the Centre for Modeling Human Disease, Toronto, and supplied by the Knockout Mouse Project (www.KOMP.org). In these cells, GFP is inserted at the *flotillin 2* locus, and GFP is expressed at low levels in *flotillin 2-/-* mice. Primers used for genotyping were: PUPA5B, GAA GCG AGA AGC GAA CTG ATT; FA2, CTT GGA AGA ATG ATG CTG TTG C; and RB4: GAG AAA GTT AGA CAT AGA GGA. Flotillin 1 knockout mice have been described previously [10].

### Ethics statement

All experiments involving animals were regulated by a Project Licence held by BN under the UK Animals Scientific Procedures Act 1986, and were approved by the Laboratory of Molecular Biology Ethical Review Committee.

### Constructs and antibodies

APP695 was a gift from Paul Matthews (CDR, Orangeburg). The Swedish mutation (K595N/M596L) was generated using site-directed mutagenesis. The N-terminal tagging of APP was obtained by introducing the fluorescent protein (GFP or mEos2) in the Kunitz-type protease inhibitor domain of APP as previously described [33].

Mouse monoclonal antibodies against flotillin 1 and 2 (BD) were used as described previously[10]. Monoclonal antibodies against amyloid beta (6E10), clathrin heavy chain (X22) and tubulin alpha (AbD Serotec) were used.

### Cell culture

HeLa cells and mouse embryonic fibroblasts were cultured in DMEM containing 10% FCS at 37°C, 10% CO2.

### Microscopy and internalisation assays

For microscopy, cells were transfected with FugeneHD (Promega), in media without Phenol red, 14–20 hours before imaging. Cells were fixed and permeabilised with either 4% parafolmadehyde in PBS followed by 0.1% Triton X-100 or with methanol at −20°C.

Internalisation of fluorescent transferrin (Invitrogen) or anti-GFP antibody (Invitrogen) were carried out in DMEM without serum at 37°C for the indicated times. Control cells were labeled for the same length of time on ice.

Pearson's correlation coefficient was calculated using the Coloc2 plugin for Image J. Images were manually adjusted to set background signal to zero, and to set maximal pixel intensity to similar values using the auto brightness and contrast function of Image J.

### Super-resolution microscopy and cluster size determination

For Photoactivation Localisation Microscopy (PALM) [34], Lab-Tek™ II chambered coverglass (Nunc) was coated with fibronectin (2 µg/ml in PBS) (Sigma Aldrich). The following day MEFs immortalised by transfection with SV40 T were seeded at a density of 6×10^3^ cells/cm^2^ and 24 h later they were transiently transfected. Approximately 16 hours after transfection, cells were fixed in 4% paraformaldehyde in PBS at room temperature. TIR-PALM imaging was performed on an Olympus IX71 microscope with a 60× 1.49NA objective (Olympus, ApoN) and an Evolve EMCCD camera (Photometrics). Activation by the 405 nm laser and excitation by the 561 nm laser were taking place at the same time. In order to distinguish photoconversion of individual molecules, the intensity of the 561 nm laser was maintained at a high level so that fluorophores would bleach within seconds after photoconversion. Typically, nonphotoconverted molecules were exhausted after 10000 frames. Centroids were assigned with the QuickPALM plugin for Fiji. The reconstructed image was then used to measure the size of the APP clusters at the plasma membrane.

### ELISA and amyloid plaque quantification

Brain tissues were harvested when mice reached the age of 12 weeks. The cerebellum and the olfactory bulbs were removed, and then the brain hemispheres were separated and individually frozen. The right hemisphere was always used for ELISA, while the left hemisphere was always used for imaging and quantification of amyloid plaques.

Brain Aβ levels from snap-frozen hemispheres were determined using Human Amyloid β40 and Amyloid β42 Brain ELISA (Millipore). Soluble Aβ was present in the supernatant after homogenization and 10 minutes centrifugation at 20,000 rcf. Insoluble Aβ, mostly in amyloid plaques, was extracted by the addition of 70% formic acid (FA) to the pellet and subsequent sonication, followed by neutralization with 1M Tris pH 7.4.

For the quantification of amyloid plaques, brain hemispheres were frozen in O.C.T. cryoprotectant compound. Subsequently, 25 µm-thick coronal sections were taken every 250 µm on a Leica CM3050S cryostat-microtome. This procedure generated approximately 25 slices from a single hemisphere. The sections were left to dry overnight and the following day were stained with Congo Red (Sigma Aldrich). Images were acquired on a Leica MZFLIII stereo microscope using the mCherry filter. Images of individual slices were then analyzed in batch with ImageJ to determine the area of amyloid plaques relative to the size of every slice.
